# Parallel processing in the brain's visual form system: an fMRI study

**DOI:** 10.3389/fnhum.2014.00506

**Published:** 2014-07-30

**Authors:** Yoshihito Shigihara, Semir Zeki

**Affiliations:** Wellcome Laboratory of Neurobiology, University College LondonLondon, UK

**Keywords:** hierarchy, early visual areas, fMRI, retinotopic mapping, parallel processing, form vision, dynamic parallelism

## Abstract

We here extend and complement our earlier time-based, magneto-encephalographic (MEG), study of the processing of forms by the visual brain (Shigihara and Zeki, 2013) with a functional magnetic resonance imaging (fMRI) study, in order to better localize the activity produced in early visual areas when subjects view simple geometric stimuli of increasing perceptual complexity (lines, angles, rhombuses) constituted from the same elements (lines). Our results show that all three categories of form activate all three visual areas with which we were principally concerned (V1–V3), with angles producing the strongest and rhombuses the weakest activity in all three. The difference between the activity produced by angles and rhombuses was significant, that between lines and rhombuses was trend significant while that between lines and angles was not. Taken together with our earlier MEG results, the present ones suggest that a parallel strategy is used in processing forms, in addition to the well-documented hierarchical strategy.

## Introduction

Ever since their discovery by Hubel and Wiesel (1959), the orientation selective (OS) cells of the primary visual cortex (area V1) have been considered to be the source of the physiological building blocks for the elaboration of forms in the visual brain (Hubel and Wiesel, 1977; Riesenhuber and Poggio, 1999; Lerner et al., 2001; Nandy et al., 2013, *inter alia*). That there is a physiological hierarchy in the properties of OS cells, with some having more complex properties than others, has encouraged a belief in the hierarchical doctrine, which supposes that forms are elaborated sequentially, with OS cells of simpler physiological properties feeding into OS cells with more complex ones, either in different layers of the same visual area (principally V1) or in successive visual areas, such as V2 and V3 (Hubel and Wiesel, 1962, 1965).

Apart from differences in complexity of the physiological properties of OS cells at successive stages (layers) in area V1 and between V1 and visual areas of the prestriate cortex, there is much anatomical and physiological evidence to support the hierarchical doctrine. The evidence comes in part from anatomy, with the predominant visual input from the retina terminating in area V1, where OS cells are first elaborated. It comes also from physiological studies which have traced a hierarchical, constructivist, relationship between simple and complex OS cells (Hubel and Wiesel, 1962, 1965; Alonso and Martinez, 1998; Martinez and Alonso, 2001; Finn and Ferster, 2007, *inter alia*). Finally, the serial connections between area V1 and areas V2 and V3 (Cragg, 1969; Zeki, 1969, 1971; Zeki and Shipp, 1988; Van Essen et al., 1986; Rockland, 1992; Burkhalter, 1993) are also consistent with a hierarchical model, in which OS cells of increasing complexity are not uniformly distributed in these visual areas but cells with more complex properties are rather preferentially distributed in areas outside V1 (Hubel and Wiesel, 1965). Mirroring this physiological hierarchy is a temporal hierarchy, reflected in the fact that cells of V1 respond with shorter latencies than cells of the prestriate visual cortex (Raiguel et al., 1989; Maunsell and Gibson, 1992; Lamme and Roelfsema, 2000).

There is however also reliable anatomical and physiological evidence to suggest that, in addition to the hierarchical strategy, the brain may also use a parallel one to elaborate forms. Anatomical evidence has established that, in addition to the inputs from V1 to prestriate areas such as V2 and V3 (Cragg, 1969; Zeki, 1969), there is a direct projection from subcortical visual nuclei such as the pulvinar and the lateral geniculate nucleus (LGN) to prestriate visual areas, including areas V2, V3, V4, and V5 (Cragg, 1969; Benevento and Rezak, 1976; Benevento and Yoshida, 1981; Fries, 1981; Yukie and Iwai, 1981; Bullier and Kennedy, 1983; Kennedy and Bullier, 1985; ffytche et al., 1995; Sincich et al., 2004; Leh et al., 2008; Baldwin et al., 2012; Cortes and Van Vreeswijk, 2012). This “V1-bypassing” input can sustain a weakened visual activity in V2 and V3 even in the absence of V1, with cells in both areas deprived of a V1 input still displaying orientation selectivity (Schmid et al., 2012). There is, as well, physiological evidence to show that OS cells with tunings similar to those in V1 but with larger receptive fields are widely distributed in the primate visual brain, and constitute heavy concentrations in V2, V3, and V3A (Zeki, 1978; Larsson et al., 2010), thus raising the question of what further contribution these cells make to the elaboration of forms. Moreover, latency studies have shown that, when appropriately tailored to the functional requirements of prestriate visual areas, the latency elicited in a prestriate area such as V5 may actually precede that in V1 (or V2) (ffytche et al., 1995; Schoenfeld et al., 2002; Gaglianese et al., 2012). This evidence made it interesting for us to investigate the possibility that a parallel strategy, *whose source is not confined to V1*, is also used by the brain to elaborate forms.

Our enquiry into a parallel contribution to the processing of forms began with a time-based magnetoencephalograpic (MEG) study (Shigihara and Zeki, 2013), which showed that two forms of increasing perceptual hierarchy—lines and rhombuses constituted from them—activate V1 and the early visual areas of prestriate cortex within the same time frame (at between 27 and 44 ms). Here, we extend that study by using functional magnetic resonance imaging (fMRI), to compensate for the relatively low spatial resolution of MEG and thus to better localize the activity produced in prestriate cortex. In doing so, we also enlarged the repertoire of forms viewed. In our MEG study, we had chosen lines because of the ubiquity of OS cells in areas V1–V3, and rhombuses consisting of the same lines because they are perceptually more complex. In this one, we added angles partly because they are intermediate in perceptual hierarchy between lines and rhombuses and partly because rhombuses have angles as constituents, which have been considered to be potent stimuli for cells in V2 (Hegdé and Van Essen, 2000; Ito and Komatsu, 2004), thus raising the possibility that they may activate human V2 more strongly than lines. Our general hypothesis, derived from our MEG studies, was that all three forms derived from straight lines will activate all the three visual areas with which we are principally concerned equally.

## Materials and methods

### Subjects

Nine right-handed healthy adult volunteers (5 female, mean age 29.2 years) took part. All had also participated in our previous study using MEG. The activity in the brains of 11 further subjects, who had also taken part in the MEG study, was also studied using the identical stimuli and paradigm except that we did not obtain retinotopic maps for them. Data from these 11 subjects are shown separately in the Supplementary Data. None of the subjects had a history of neurological or psychiatric disorder; written informed consent was obtained from all and the study was approved by the Ethics Committee of University College London.

### Stimuli and task

**Figure 1:**
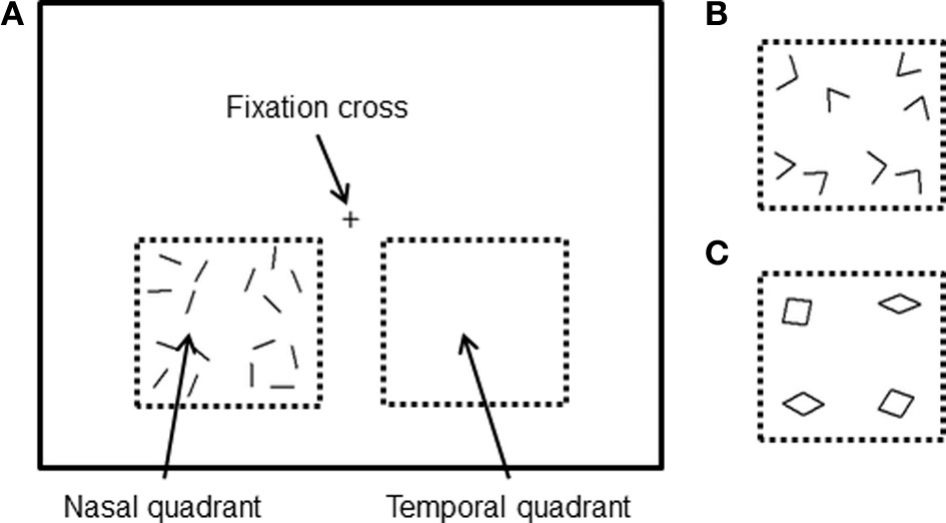
**Stimuli were viewed using the right eye only and were presented in the lower region of the visual field in either the nasal or temporal quadrant**. All stimuli consisted of 16 white lines, presented either separately **(A)**, joined to form eight angles **(B)** or joined to form four rhombuses **(C)**.

In general, we tried to keep our stimulus presentation and paradigm as similar to the ones we used in our MEG study, to allow for a direct comparison. The stimuli were generated in the same way as in our previous MEG study (Shigihara and Zeki, 2013) but differed in (a) the addition of a further category (angles) constituted from the same lines and intermediate in perceptual complexity between lines and rhombuses and (b) a reduction in the size of all the form stimuli by 14%, because of the smaller fMRI scanner screen size. To match our previous study MEG study, subjects viewed the fixation cross (subtending 0.9 × 0.9°) at the center of the screen monocularly with the right eye (a patch covered the left eye). All stimuli were displayed separately in either the lower left (nasal) or lower right (temporal) quadrants of the visual field, between 1.1 and 10.5° below the fixation cross and 1.9–11.8° on either side. Three different form stimuli were used: 16 lines, 8 angles, or 4 rhombuses (Figures 1A–C), all consisting of the same16 white lines. The vertices of angled stimuli varied from 54 to 108° and those of rhombuses from 18 to 162°. The same image (e.g., oriented lines) flashed at 8 Hz (67 ms between flashes) without changing the orientation of the line, to maximize the activation during the scans (Kwong et al., 1992); it continued to flash for 4 s, then disappeared and was replaced by a new stimulus which could be another oriented line or one of the two other categories (angles and rhombuses), the sequence being randomized.

There were four 5-min runs; each had 63 stimulus periods of 4 s duration. After every 21 stimuli there was a 20 s rest period, which served as baseline. The sequences of six different conditions (2 quadrants × 3 forms) were pseudo-randomized and counterbalanced across runs and participants. To reduce participants' eye movements and maintain their attention, the fixation cross periodically increased its vertical size from 0.9 to 1.1° for a brief interval (100 ms), which subjects were asked to report by pressing a button with their right index finger.

### Scanning details

Scanning was done in a 3.0-T Siemens Magnetron Allegra MRI scanner (Siemens, Erlangen, Germany). There were two scanning sessions, functional and retinotopic, the latter to identify the borders between V1, dorsal V2, and dorsal V3 (V2d and V3d, respectively) since we presented our stimuli in the lower quadrants only. Each run of the functional and retinotopic mapping sessions began with a blank screen (black for the functional session, gray for the retinotopic) for ~17 s; the first six brain volumes acquired were subsequently discarded to allow for T1 equilibration effects. An echo-planar imaging (EPI) sequence was applied to obtain Blood-oxygen-level dependent (BOLD) signal (echo time TE = 30 ms, repeat time TR = 2.88 s) using 48 slices to cover the whole brain. The voxel resolution was 3 × 3 mm in-plane resolution, with a 2 mm slice thickness and 1 mm inter-slice gap. A T1-weighted anatomical image was acquired for each subject (176 slices, resolution 1 × 1 × 1 mm, TE = 2.48 ms, TR = 7.92 ms) between those scans.

### Functional sessions

Functional images were pre-processed and analyzed using SPM-8 (http://www.fil.ion.ucl.ac.uk/spm). They were realigned to the first volume of the first experimental session, and re-sliced to a final voxel resolution of 3 × 3 × 3 mm^3^ to match the functional and retinotopic scans because, for some subjects, the two scans were performed on separate days. Normalization and smoothing were not applied because the data are based on individual brains. The stimulus for each subject was modeled as a set of regressors in a general linear model (GLM). Boxcar function was used to define stimulus functions, which modeled the onsets and durations of each stimulus period. Head movement parameters calculated from the realignment pre-processing step were included as regressors of no interest. Stimulus functions were convolved with a canonical Hemodynamic Response Function to provide regressors for the GLM. Contrast images for each form vs. baseline were made and used to evaluate average activity in each visual area (described below).

### Retinotopic mapping

**Figure 2:**
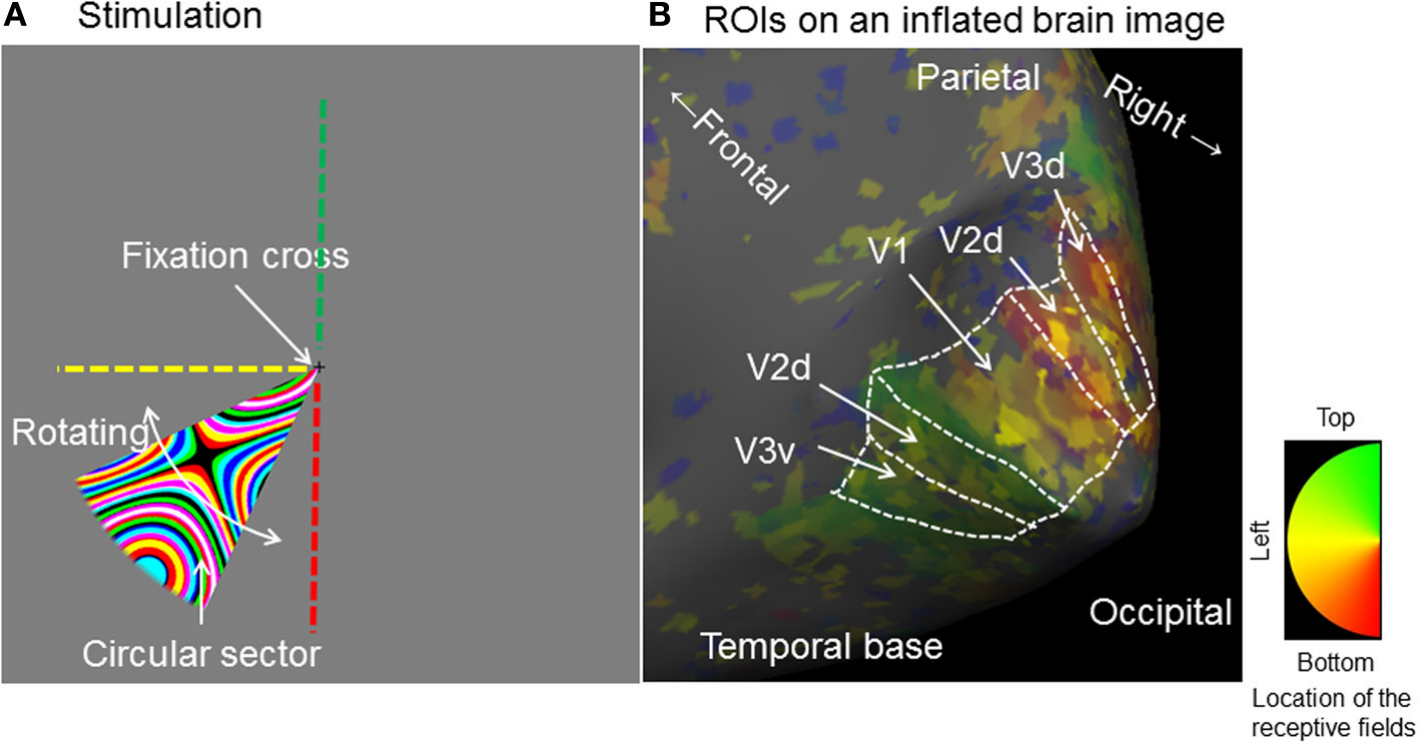
**Stimulation for obtaining retinotopic maps and charting region of interest on an inflated brain image for a typical single subject. (A)** Stimulus: A circular sector (central angle 40°) sustaining 8° and rotated in either a clockwise or anticlockwise direction for eight cycles at an angular velocity of 61.2 seconds per cycle. The sector was filled with a dynamically expanding colored ripple pattern. Green, yellow and red broken lines indicate the upper vertical left horizontal and lower vertical lines of the screen. **(B)** Retinotopicaly identified ROIs for V1–V3 on an inflated anatomical brain image (Right hemisphere): The colored overlay image is a phase map which represents the brain area responding to the stimulus location on the screen. The borders for each ROI were manually identified. d, dorsal; v, ventral.

In this study, we are especially interested in three early visual areas: V1, V2d, and V3d; we used retinotopic mapping techniques to identify their borders and make region of interest (ROI) masks to cover them. The scan for retinotopic mapping consisted of two 8 min runs, one with a clockwise and the other an anti-clockwise stimulus direction. Individual retinotopic maps were obtained from all nine subjects because of individual differences in the position and borders of early visual areas (Stensaas et al., 1974). Retinotopic mapping stimuli were generated with scripts written by Schwarzkopf (http://www.fil.ion.ucl.ac.uk/~sschwarz/retinotopy.html) for MATLAB and Psychtoolbox-3 (http://psychtoolbox.org/HomePage) and using the same projector as the functional stimuli. A rotating wedge-shaped stimulus containing an expanding color ripple pattern was used (Figure 2A). To make ROI masks for areas V1, V2d, and V3d in each subject, EPI images from the retinotopic sessions were transformed to phase space (phase maps) using phase-encoded retinotopic mapping techniques (Sereno et al., 1995) and averaged with scripts written by Schwarzkopf (http://www.fil.ion.ucl.ac.uk/~sschwarz/retinotopy.html). These phase maps were then overlaid onto an inflated anatomical brain image which had been created individually for each subject using FreeSurfer (http://surfer.nmr.mgh.harvard.edu/). The boundaries of V1, V2d, and V3d were then manually delineated by identifying the representation of the meridians from the mirror reversals in the phase map (Figure 2B). As our interest was focused on V1, V2d, and V3d we did not extend our retinotopic mapping to areas V3A and V3B.

### Evaluating average activation intensity in each visual area (ROI analysis)

The average activation intensity of all voxels in each of the three visual areas of the contralateral hemisphere was obtained from the results of the contrast images (each form vs. baseline) and from ROI masks (V1, V2d, and V3d) during the retinotopic session for each subject, further broken down by form and quadrant. A three-way (area × form × quadrant) repeated measures ANOVA was applied, followed by *post-hoc t*-tests. The Ryan method (Ludbrook, 1991) was used to make multiple comparisons. To confirm that all three forms activate all three areas, a one sample *t*-test was used after combining activation intensities across areas and quadrants, since a three-way repeated measures ANOVA had shown no main effect of area and quadrant and no interactions. *P*-values were two-tailed, with *P*-values less than 0.05 considered significant.

## Results

### Retinotopic mapping

The vertical meridian is represented at the border between V1 and V2, a representation of the horizontal meridian demarcates the border between V2 and V3 while the vertical meridian is represented again at the anterior border of V3, in both monkey and human brains (Cragg, 1969; Zeki, 1969; Shipp et al., 1995; Tootell et al., 1997, Figure 2). We were able to identify these borders in all subjects with our retinotopic mapping, and thus locate the three areas that we are concerned with retinotopically. This allowed us to learn whether each area was activated by the three categories of form stimuli used.

### Activation by the three forms

**Figure 3:**
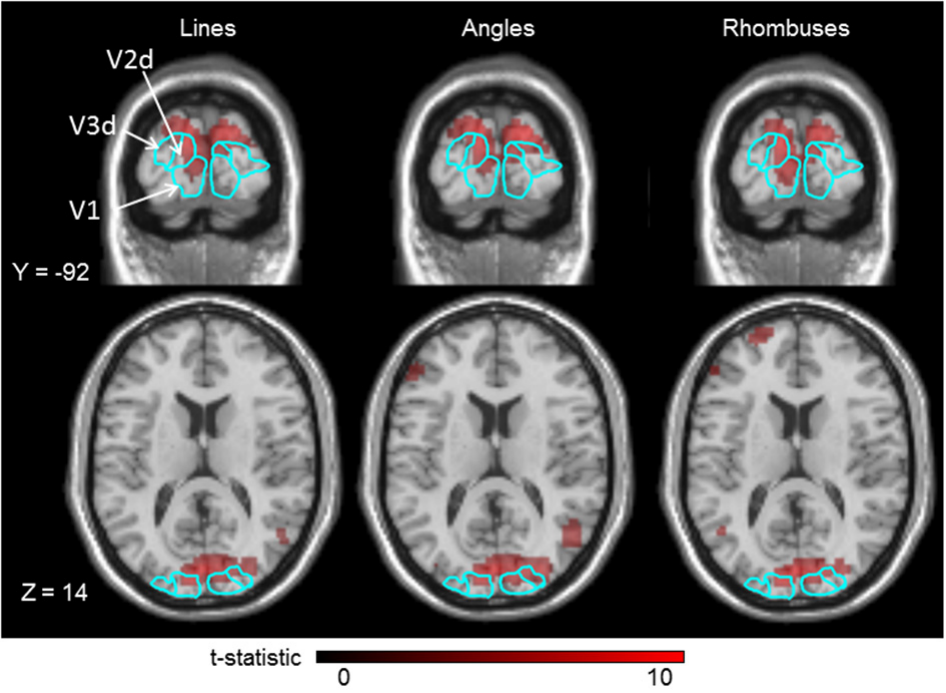
**Cortical activations produced in the three visual areas (V1–V3) by lines, angles, and rhombuses vs. baseline, for a typical single subject, superimposed on his anatomical brain image**. Smoothing was not applied. Borders of those visual areas are displayed in light blue lines. Displayed threshold of *P*(FWE) < 0.05.

A typical activation map is shown in Figure 3. The activation produced by each category of form stimulus covered all three visual areas (V1-V2d-V3d).

**Figure 4:**
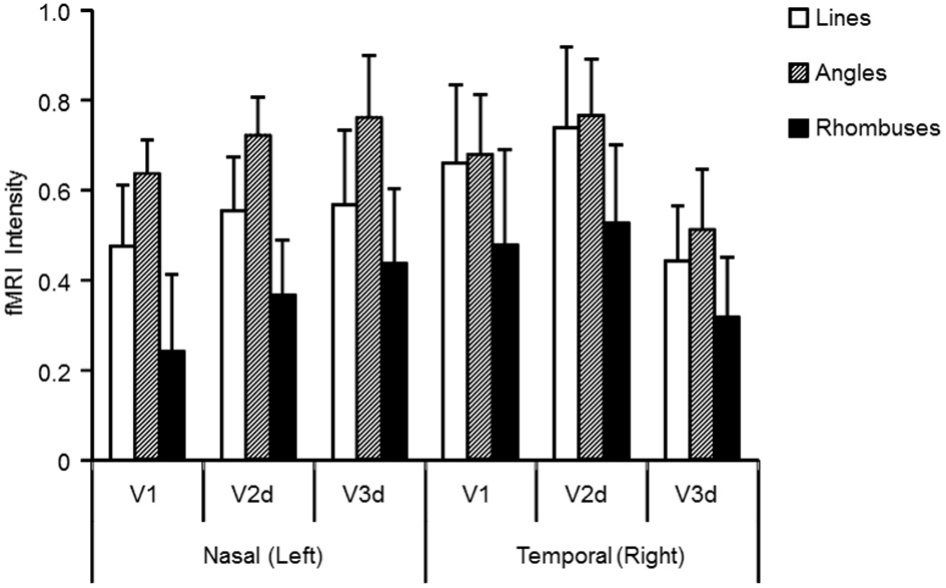
**Activation levels (averaged across nine subjects) in visual areas V1, V2d, and V3d for lines, angles, and rhombuses presented to either the nasal or the temporal visual quadrant**. Visual areas were mapped retinotopically in each subject. Standard errors of the mean are indicated by error bars. White, shaded, and filled columns indicate activation level for lines, angles, and rhombuses respectively, derived from the BOLD signal.

To evaluate the intensity of activation in each of the three visual areas, we calculated the intensity of activation within each retinotopically mapped area (Figure 4 and Table 1). One sample *t*-tests showed that all three forms produced significant activations relative to baseline in each area (Table 1: each form: *P* < 0.05). To determine which factors (quadrant, visual area, and form) modulate the intensities of activations, we employed a three-way repeated measures ANOVA (quadrant × area × form: Table 2). This showed a main effect of form, but not of area or quadrant and no interactions. Hence there were differences in average activation produced by lines, angles and rhombuses but none in average activation between visual areas or in the visual quadrant (lower nasal or temporal) stimulated. In terms of form, *post-hoc t*-tests showed that angles produced significantly stronger activations than rhombuses (*P* = 0.006) for all three areas. The difference between the activity produced by angles and rhombuses was trend significant (*P* = 0.066) while that between lines and angles was not (*P* = 0.254) (Table 2). In summary, all three forms activated all three areas and angles produced the strongest activation in each visual area.

**Table 1:** **Results of one sample *t*-test comparisons of Blood-oxygen-level dependent signal intensities associated with areas and forms across nine subjects**.

**Area**	**Form**	**Statistics**	***P***
V1	Line	*t*_(17)_ = 5.158	7.89E-05
	Angle	*t*_(17)_ = 9.054	6.50E-08
	Rhombus	*t*_(17)_ = 2.675	1.60E-02
V2d	Line	*t*_(17)_ = 6.044	1.32E-05
	Angle	*t*_(17)_ = 10.211	1.14E-08
	Rhombus	*t*_(17)_ = 4.246	5.45E-04
V3d	Line	*t*_(17)_ = 5.078	9.32E-05
	Angle	*t*_(17)_ = 6.554	4.91E-06
	Rhombus	*t*_(17)_ = 3.611	2.16E-03

Blood-oxygen-level dependent signal intensities (vs. baseline) were averaged within each visual area in contra-lateral hemisphere and were combined across quadrants. A one sample t-test showed that all three forms produced significant activations in all areas.

**Table 2:** **Results of Three-Way ANOVA comparisons of Blood-oxygen-level dependent intensities associated with quadrants, areas and forms across nine subjects**.

		**Statistic**	***P***
Main effect	Quadrant	*F*_(1, 8)_ = 0.136	7.22E-01
	Area	*F*_(2, 16)_ = 0.822	4.57E-01
	Form	*F*_(2, 16)_ = 5.092	**1.94E-02**
Interaction	Quadrant-area	*F*_(2, 16)_ = 2.114	1.53E-01
	Quadrant-form	*F*_(2, 16)_ = 1.030	3.80E-01
	Area-form	*F*_(4, 32)_ = 0.625	6.48E-01
	Quadrant-area-form	*F*_(4, 32)_ = 0.462	7.63E-01
*Post-hoc*	Angle > rhombus	*t* = 3.158	**6.09E-03**
	Angle > line	*t* = 1.184	2.54E-01
	Line > rhombus	*t* = 1.974	6.59E-02

Blood-oxygen-level dependent intensities (vs. baseline) were averaged within each area in the contra-lateral hemisphere. A Three-Way ANOVA showed that forms modulate averaged intensities. Post-hoc t-test showed that the averaged intensity for angles was significantly stronger than for rhombuses regardless of quadrants and areas. Bold digits indicate significant difference (P < 0.05).

### Additional scans

All 20 subject who were enrolled in our previous MEG study (Shigihara and Zeki, 2013) were also scanned functionally. The details are shown in the Supplementary data. In brief, whether we use 9 or 20 subjects, group analysis shows that all three forms activated both striate and prestriate cortices and that the strengths of activation produced by the three forms were similar.

## Discussion

Areas V2 and V3 of the primate visual brain surround area V1, generally considered to be the first and most prominent recipient of visual signals in the cortex, and are prominently connected with it (Cragg, 1969; Zeki, 1969, *inter alia*). This anatomical arrangement as well as the sequence of latencies in the areas provoked by visual stimulation (Lamme and Roelfsema, 2000 for a review) reinforces support for the hierarchical doctrine of form perception, which supposes that OS cells of V1 are the source of the physiological building blocks of form, from which further, more complex, forms are elaborated. As we point out in the Introduction, there is much to support such a claim. But, in the work reported here, we set out to extend our earlier time-based study using MEG (Shigihara and Zeki, 2013), to learn whether there is not, in addition, a parallel strategy in the construction of form involving these three areas. The results reported here, taken together with our earlier MEG evidence, lead us to suspect that a parallel strategy, whose source may not be confined to V1, may also be used to elaborate forms, in addition to the well-documented hierarchical strategy.

### Parallel and hierarchical strategies

It is perhaps instructive to begin by laying out what result, given our stimuli and the paradigm we have used, the hierarchical and parallel strategies would predict.

A strictly hierarchical strategy would predict that three visual areas with which we are principally concerned (V1–V3) should not be activated with the same strength by all the three forms. Instead, one might expect rhombuses to produce the strongest response, since they have the constituent oriented lines plus a more complex form built from them, while simple oriented lines might be expected to produce the weakest activity. Alternatively, if there is an uneven distribution of cells of differing complexity between the three areas (Hubel and Wiesel, 1965), one might expect that the simplest form—oriented lines—will activate the “earlier” visual area, V1, more strongly while rhombuses will activate “later” visual areas (e.g., V3) more strongly. As well, the hierarchical strategy would predict that the latency of activation should be briefest for simple lines and longest for the perceptually more complex rhombuses, with angles falling somewhere in between.

By contrast, the parallel model would predict that (a) all three forms should activate all three visual areas and that each form should do so with more or less equal strength; (b) that the three different forms should activate the cortex with similar latencies.

In fact, the results we obtained support and give credence to the utilization of a parallel strategy to construct forms. All three forms activated all three visual areas, each form activating each visual area with the same strength although, overall, angles produced the strongest activation in all three areas and rhombuses the weakest. These results are consistent with our earlier, time-based results (Shigihara and Zeki, 2013) which showed that (a) lines and rhombuses produced very early responses (between 27 and 44 ms) in occipital areas; (b) the sources of the responses were estimated (localized) in both striate (V1) and prestriate cortices; (c) in the MEG study, the response amplitude for lines was stronger than that for rhombuses, implying a more powerful activation produced by lines than by rhombuses, which is the opposite of what one might expect from an exclusive hierarchical strategy. When we say that this evidence supports the possibility that a parallel strategy is also used to construct forms, we do so without implying that a hierarchical strategy is not used. Our principal and sole aim here was to explore the extent to which a parallel strategy may be used to construct forms; it was not aimed at documenting the evidence in favor of a hierarchical strategy, which has been documented many times, or to determine which of the two strategies is the more potent one.

### Parallel visual inputs to V1 and prestriate cortex: evidence from anatomy and latency studies

That there are parallel strategies within visual cortex itself, implied by the parallel anatomical connections from V1 and V2 (which are themselves interconnected) to, say, V4 and V5 (Zeki and Shipp, 1988), has long been acknowledged and its computational significance evaluated (e.g., Zeki, 1976; Ballard et al., 1983; Grossberg, 1991). Much less attention has been given to parallel inputs to V1 and areas of the prestriate cortex from sub-cortical visual stations such as the LGN and the pulvinar, though it has received some recently (Cortes and Van Vreeswijk, 2012). This is surprising since such pathways have been known to exist for a relatively long time from LGN (Cragg, 1969; Fries, 1981; Yukie and Iwai, 1981) and pulvinar (Cragg, 1969; Benevento and Rezak, 1976; Baldwin et al., 2012), and their capacity to mediate a crude but conscious experience of vision acknowledged (Barbur et al., 1993; Sahraie et al., 1997; Zeki and ffytche, 1998; Schoenfeld et al., 2002). Perhaps this neglect is due to the dominant role played by the classical geniculate to V1 pathway, lesions in which lead to blindness commensurate with the size and position of the lesion. Yet there is also evidence that lesions restricted to V2 and V3 lead to a comparable blindness (Horton and Hoyt, 1991), although such evidence is sparse because much more difficult to obtain due to the disposition of V2 and V3 in relation to V1, which means that damage to the latter usually also involves damage to the former. Perhaps it is due as well to latency studies, which have commonly used flash stimuli to evoke responses from cortex and which, collectively, have shown that activity in V1 precedes that in prestriate visual areas (see Lamme and Roelfsema, 2000 for a review). But when stimuli are better tailored to the properties of individual areas of the prestriate cortex, a more complex picture emerges, in which prestriate areas may receive visual input earlier than V1, depending on the nature of the stimulus, as in the example of V5 (ffytche et al., 1995; Gaglianese et al., 2012). The dual input to V5 from the sub-cortex (ffytche et al., 1995; Sincich et al., 2004), one mediated through V1 and the other by-passing V1 and terminating directly in V5, was demonstrated by using stimuli that differed in speed, with fast moving stimuli activating V5 before activating V1, leading to the concept of a *dynamic parallelism* (Beckers and Zeki, 1995; ffytche et al., 1995). Hence, it becomes plausible to suppose that these direct inputs to visual areas of the prestriate cortex with large concentrations of OS cells may deliver signals related to form vision directly to V2 and V3 and V3A, without passing through V1 (Schmid et al., 2012), just as they deliver motion-related signals directly to V5 (ffytche et al., 1995; Schoenfeld et al., 2002; Sincich et al., 2004). Once again, we emphasize that a parallel system must be integrated with the hierarchical system; this is indeed implicit in the demonstration that, although cells in V2 and V3 are reactive to the appropriate visual stimuli in the absence of V1, the strength of activity in them is significantly reduced (Schmid et al., 2012).

### Limitations of our study

#### Stimuli

There are many different ways of stimulating the visual form system and we do not pretend to have used the optimal stimuli for activating all three visual areas, V1–V3, in this study. We could, for example, have used pictures of real objects or Glass patterns (Ostwald et al., 2008). We tailored our stimuli as best we could to the reported physiology of these areas, which shows that all three contain heavy concentrations of OS cells (Zeki, 1978; Larsson et al., 2006, 2010). More generally, our concern was mainly with OS cells and whether an exclusive hierarchical strategy is used to integrate their responses into more elaborate forms, sequentially and hierarchically.

Our stimuli nevertheless reveal an interesting feature, which may yet turn out to be significant. Although we obtained a significant difference in strength of activity produced by angles and rhombuses, the difference in strength between lines and angles was not significant, in that they both activated the three visual areas with similar strengths. This is mirrored in psychophysical masking studies (Lo and Zeki, 2014), in which we found that angles and rhombuses do not mask each other significantly (implying independent processing of the two, see Cheadle and Zeki, 2011) while lines and angles do. This implies that lines and angles may belong to the same family of forms, distinct from the more complex rhombuses, even if—as our current evidence suggests—angles and lines may be processed in parallel within the three visual areas.

#### Possible confounds due to the density of the stimuli

A possible confound in our results relates to differences in stimulus density between lines, angles and rhombuses. Our stimuli were evidently not equal in this respect. A previous study has shown that neural responses can be modified by stimuli consisting of lines of different orientation outside of the cells' receptive fields (Knierim and Van Essen, 1992). However, if so, then angles and rhombuses should produce a stronger response than lines. But in our study rhombuses produced weaker responses than angles so this effect cannot be critical for this study. We also discount the possibility that factors such as the spatial frequency of our stimuli (Singh et al., 2000) or “explaining away” (Kersten et al., 2004) might account for why rhombuses produced relatively weaker responses. The spatial frequency profile of our stimuli shows that rhombuses had the highest spatial frequency components, angles intermediate ones, lines and the lowest (See Supplementary Data). Hence spatial frequency cannot explain our results because the order of spatial frequency profiles for the three forms does not match the order of the activation strength.

#### Temporal hierarchy

It is also worth outlining another limitation of our study which precludes us from any strong statements regarding the use of a parallel strategy. In terms of the latency of activation reported in our previous study (Shigihara and Zeki, 2013), we found that all three areas were activated within the same time frame of 27–44 ms when subjects viewed lines and rhombuses. This compares with early latencies of 30–50 ms for activation of V1 reported in the monkey (Maunsell and Gibson, 1992). Although the use of dynamic causal modeling (DCM) for these results strongly favored the parallel model over the hierarchical one (see Shigihara and Zeki, 2013), there still remains the possibility that our results did not detect, for technical reasons, much smaller differences in latency—in the 5–10 ms range, in activation between the three areas. It is therefore possible that an area such as V1 may have been activated by, say, 10 ms before an area such as V3. But in light of the evident overlap in latency of activation between these areas and the activation of V1 at 30–50 ms, we must still consider similar latencies of activation as a strong pointer to the possibility of parallel processing.

### Parallel strategies in form perception: evidence from psychophysics

The psychophysical results of Li and Gilbert (2002) and Bell et al. (2011) have shown that there may be intermediate stages to form perception even in early visual areas, including area V1 (see review by Loffler, 2008). Our results are consistent with this and suggest that the early and intermediate stages may occur in parallel within each of the three visual areas (V1–V3). On the other hand, imaging studies show that all areas extending from V1 to lateral occipital complex (LOC) respond well to global forms, leading to the suggestion that they all “integrate local elements to global shapes” (Kourtzi et al., 2003). We believe in light of our present results that they may do so not only hierarchically but also in parallel.

Taken together, these results suggest that the perceptual hierarchy of relatively simple forms is not mirrored by a strictly sequential neurological hierarchy as far as the time course of activation of areas V1–V3 (Shigihara and Zeki, 2013) or the strength of activation produced in them by stimuli of increasing complexity is concerned. It is now well established that a hierarchical process occurs within V1 and V2 (see for example, Alonso and Martinez, 1998; Martinez and Alonso, 2001). It would thus seem that such a hierarchical strategy for the processing of forms must be used in parallel in each of the three areas that we have studied here, and possibly in other areas of the human brain that have been shown to respond well to oriented lines and that have been implicated in form perception (Vanduffel et al., 2002; Zeki et al., 2003; Fang et al., 2005; Larsson et al., 2010; McDonald et al., 2010). Recent results suggest that a parallel strategy is also used in the object-selective lateral occipital area (Silson et al., 2013). There remains the puzzle of why it is that angles produced the strongest activation in each of the three visual areas, a problem for which we have no ready answer.

We are of course also left with the puzzle of why apparently similar processes should be used in three separate visual areas, as far as can be determined from our relatively simple stimuli. One would naturally assume that they serve ultimately different ends, but what these may be is not clear at present and must remain conjectural, as must the exact contribution that each area makes to the construction of simple forms. Overall, it would be interesting to investigate what the role of each of the two strategies—the hierarchical and the parallel—is in elaborating forms and how the two co-operate to generate forms.

## Funding

The study was funded by the Wellcome Trust, London, UK. The funding bodies did not play a role in the design, execution, or analysis of the study.

### Conflict of interest statement

The authors declare that the research was conducted in the absence of any commercial or financial relationships that could be construed as a potential conflict of interest.
