# Supplementary material for: Parallel processing in the brain's visual form system: an fMRI study
Source: Front Hum Neurosci. 2014 Jul 30;8:506. doi: 10.3389/fnhum.2014.00506 (PMC4115635; doi:10.3389/fnhum.2014.00506)
Supplement: Supplementary file 1 [file DataSheet1.DOCX]

**Supplementary data**

1. **Categorical analysis for 20 subjects**

Here we report the results from a larger sample, where we include 11 more subjects, in addition to the nine subjects in the main study. These extra subjects completed the same functional sessions as the main group but did not undergo retinotopic mapping procedures. The material and methods used are identical to those described in the main text, except that here we have 20 subjects (10 female, mean age 28.2 years), although we obtained retinotopic maps only for the 9 subjects described in the main text. All the subjects described here were also involved in the MEG study, published in Shigihara and Zeki (2013).

The results show that a three forms activated all three visual areas (striate and prestriate cortex). Contrasts for each of the three forms vs baseline (Figure S1 and Table S1) revealed significant activations produced by all three forms.

**
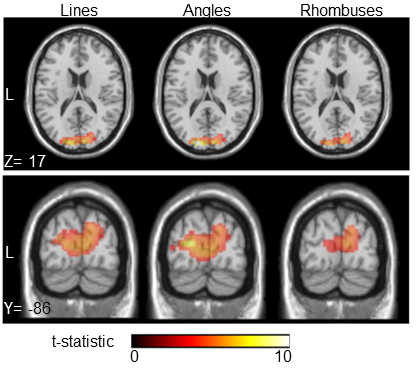
**

**Figure S1.**

Cortical activations produced in the three areas by lines, angles and rhombuses versus baseline, across 20 subjects, superimposed on coronal and horizontal sections of a canonical brain. Displayed threshold of P(unc.) < 0.001.

**Table S1. MNI co-ordinates and significance of fMRI activations versus baseline.**

|  | X | Y | Z | Visual area | P(FWE) (cluster) | Cluster  size |
| --- | --- | --- | --- | --- | --- | --- |
| Lines > Baseline | -12 | -91 | 13 | Prestriate | 1.11E-08 | 766 |
| Angles > Baseline | -15 | -91 | 19 | Prestriate | 5.97E-09 | 871 |
| Rhombuses > Baseline | 12 | -91 | 13 | Prestriate | 3.34E-06 | 415 |
|  | -39 | -70 | 10 | Prestriate | 2.96E-02 | 92 |

1. **Spatial frequency analysis**

To investigate the effects of differences in spatial frequency in our three stimuli we carried out the following analysis.

We took ten bitmap image stimuli from each category (lines, angles and rhombuses) as used in our functional scans, but excluding the fixation cross.

The two-dimensional discrete fourier transform of each image was calculated using the Matlab fft2 command. The intensities of the spatial frequency of the two axes (horizontal and vertical) were added together to give a spatial frequency spectrum and then averaged for each of the three categories (Figure S2).

All three curves show similar patterns: they decrease in intensity as frequency increases and each curve has a small peak at around 5 c/degree. The peak frequency for rhombuses is higher than the others but those for lines and angles are almost the same. For the higher component frequencies (10-20 c/degree) rhombuses have the strongest, angles have intermediate and lines have the weakest intensity. Frequencies below 3 c/degree show no clear differences between the three types of stimuli. In summary, we can say that at higher frequencies rhombuses are the strongest and lines are the weakest.


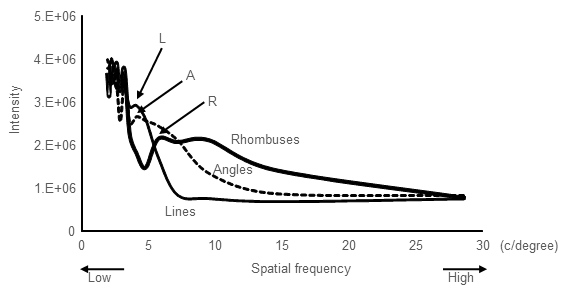


Figure S2. Spatial frequencies of the stimuli for three categories. Arrows with small letters show small peaks of curves. L, lines; A, angles; R, rhombuses.

Conclusion:

Our fMRI analysis showed that angles produced the strongest and rhombuses the weakest activation in all three visual areas. Ranking the three categories by intensity of activation (i.e. Angles > Lines > Rhombuses) does not match the ranking by spatial frequencies (i.e. Rhombuses > Angles > Lines for higher frequencies or no clear distinction for low frequencies). We therefore conclude that spatial frequency is unlikey to account for our results.

**Reference**

Shigihara, Y., and Zeki, S. (2013) Parallelism in the brain’s visual form system. *Eur. J. Neurosci.* 38, 3712–3720.
