# Supplementary figures and images for: Parallel processing in the brain's visual form system: an fMRI study
Source: Front Hum Neurosci. 2014 Jul 30;8:506. doi: 10.3389/fnhum.2014.00506 (PMC4115635; doi:10.3389/fnhum.2014.00506)

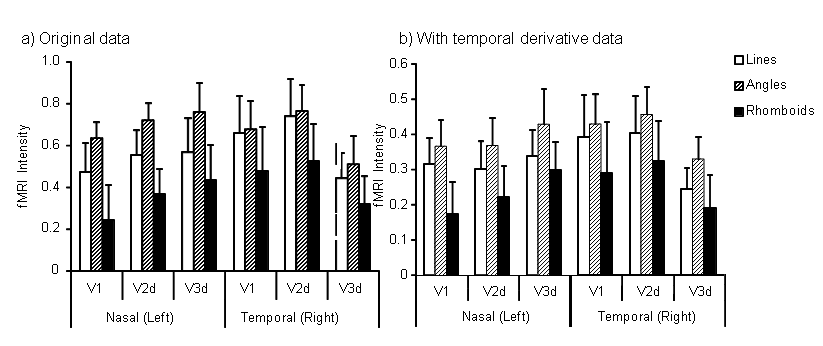


Figure 1R. Activation levels calculated without and with temporal derivative

Supplement: Supplementary file 2 [file DataSheet2.DOCX]
